# Independent predictors of breast malignancy in screen-detected microcalcifications: biopsy results in 2545 cases

**DOI:** 10.1038/bjc.2011.466

**Published:** 2011-11-03

**Authors:** G Farshid, T Sullivan, P Downey, P G Gill, S Pieterse

**Affiliations:** 1 BreastScreen SA and SA Pathology, 1 Goodwood Road, Wayville, South Australia 5034, Australia; 2 Data Management and Analysis Centre, Discipline of Public Health, University of Adelaide, Adelaide, Australia

**Keywords:** breast cancer, screening, mammography, microcalcifications

## Abstract

**Background:**

Mammographic microcalcifications are associated with many benign lesions, ductal carcinoma *in situ* (DCIS) and invasive cancer. Careful assessment criteria are required to minimise benign biopsies while optimising cancer diagnosis. We wished to evaluate the assessment outcomes of microcalcifications biopsied in the setting of population-based breast cancer screening.

**Methods:**

Between January 1992 and December 2007, cases biopsied in which microcalcifications were the only imaging abnormality were included. Patient demographics, imaging features and final histology were subjected to statistical analysis to determine independent predictors of malignancy.

**Results:**

In all, 2545 lesions, with a mean diameter of 21.8 mm (s.d. 23.8 mm) and observed in patients with a mean age of 57.7 years (s.d. 8.4 years), were included. Using the grading system adopted by the RANZCR, the grade was 3 in 47.7% 4 in 28.3% and 5 in 24.0%. After assessment, 1220 lesions (47.9%) were malignant (809 DCIS only, 411 DCIS with invasive cancer) and 1325 (52.1%) were non-malignant, including 122 (4.8%) premalignant lesions (lobular carcinoma *in situ*, atypical lobular hyperplasia and atypical ductal hyperplasia). Only 30.9% of the DCIS was of low grade.

Mammographic extent of microcalcifications >15 mm, imaging grade, their pattern of distribution, presence of a palpable mass and detection after the first screening episode showed significant univariate associations with malignancy. On multivariate modeling imaging grade, mammographic extent of microcalcifications >15 mm, palpable mass and screening episode were retained as independent predictors of malignancy. Radiological grade had the largest effect with lesions of grade 4 and 5 being 2.2 and 3.3 times more likely to be malignant, respectively, than grade 3 lesions.

**Conclusion:**

The radiological grading scheme used throughout Australia and parts of Europe is validated as a useful system of stratifying microcalcifications into groups with significantly different risks of malignancy. Biopsy assessment of appropriately selected microcalcifications is an effective method of detecting invasive breast cancer and DCIS, particularly of non-low-grade subtypes.

Microcalcifications are one of the main categories of abnormalities detectable by mammograms. The widespread uptake of screening mammography has been associated with a marked increase in the diagnosis of ductal carcinoma *in situ* (DCIS), as this lesion is frequently associated with dystrophic calcifications, particularly when comedo necrosis is present. At present, DCIS constitutes one in five screen-detected malignancies in our population-based breast cancer-screening programme.

In addition to malignat lesions, many non-malignant processes also produce dystrophic microcalcifications in breast tissue. In order to minimise invasive investigations, radiologists take into account the various characteristics of microcalcifications. The form, size, pleomorphism and distribution of the calcifications provide clues to the underlying pathology. Mammographic grading systems encapsulate the overall level of concern. The grading system used throughout Australia and parts of Europe is a five tier system that differs from the BI-RADs system used in North America (National Breast Cancer Centre, 2002). In this system, grade 3 lesions are regarded as indeterminate/equivocal and lesions graded 3 and above require imaging workup and possible biopsy.

In this study, we wished to review the outcomes of assessment of microcalcifications detected through screening mammography at our programme, with a particular interest in evaluating the discriminating capacity of the mammographic grading system used.

## Materials and Methods

### The design of our breast cancer-screening programme

BreastScreen South Australia is part of a national breast cancer-screening programme and has been accredited to provide this service since 1991, after a pilot in 1989. The design of this programme has been described previously (Farshid and Rush, 2004). In brief, asymptomatic women aged 50–69 years are invited to participate at 1 or 2 yearly intervals, depending on family history. Two radiologists read two view screening mammograms independently. A third reader arbitrates discordant results. All imaging was based on film screen mammography for the study period. A 5-tier grading scheme is applied (National Breast Cancer Centre, 2002), grade 1, normal; grade 2, benign; grade 3, indeterminate/equivocal, grade 4, suspicious for malignancy and grade 5, radiologically malignant. Women with grade 1 or 2 lesions are cleared and are invited to return for re-screening in 1–2 years. The remaining lesions are recalled for further assessment. Based on workup mammography and, in most cases, ultrasound examination, the lesion is re-graded. Some lesions are cleared as a result of these additional imaging tests. Needle biopsy is performed for those that have imaging grades 3 and above.

Lesions with malignant needle biopsy findings are referred for treatment. Diagnostic open biopsy is used when the imaging and needle biopsy findings are discordant or indefinite. For lesions undergoing surgery, all final pathology reports and treatment data are audited and are entered prospectively into an electronic database. Follow-up of clients who were not referred for surgery is through tracking them during subsequent screening visits and also via the State Cancer Registry, which is required to notify screening programmes of any cancers diagnosed in their clients within 27 months of a negative screen (interval cancers; BreastScreenAustralia, 2005). Treating surgeons and general practitioners also notify us of any interval cancers.

### Study design

We searched our prospectively accrued electronic archives for lesions screened during the period January 1992 to December 2007, in which the dominant radiological abnormality was coded as microcalcifications. We restricted inclusion to those cases in which the only imaging abnormality was microcalcifications, without other associated abnormalities, such as a mass on mammography or ultrasound. To simplify analysis, cases with more than one mammographic lesion are excluded. For each lesion, we tabulated patient demographics, imaging grade, mammographic extent, pattern of distribution of microcalcifications, biopsy methods and final outcome.

For lesions that were not excised, we obtained follow-up information by tracking the clients in our own database during their subsequent screening visits and through notifications of breast cancer diagnoses from the State Cancer Registry.

### Radiological characteristics

In our programme, in accordance with the criteria set by the Royal Australian and New Zealand College of Radiologists (National Breast Cancer Centre, 2002), we designate microcalcifications as grade 5 if they exhibit typical malignant features of distribution and morphology:

(i) Distribution: - clustered or, - aligned (separated calcifications contained  within a duct).
(ii) Morphology: - intraluminal (elongated calcifications with or  without branching). - granular (significant variability in size and  shape).

**Figure 1:**
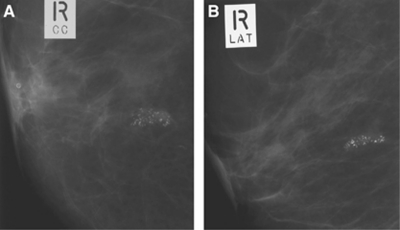
Screening mammogram showing high-grade (grade 5) microcalcifications. (**A**) Cranio-caudal and (**B**) lateral views. Note: Linear calcifications of casting morphology.

**Figure 2:**
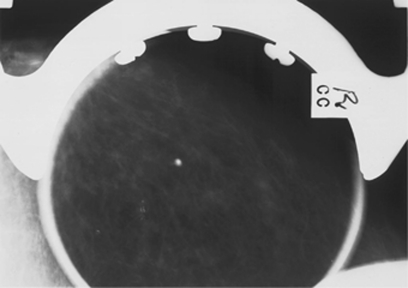
Grade 4 microcalcifications. A new lesion in the current round. Although the microcalcifications have somewhat smooth margins, they have a linear configuration with possible branching forms. The findings were regarded as suspicious for DCIS. This was confirmed by core biopsy.

**Figure 3:**
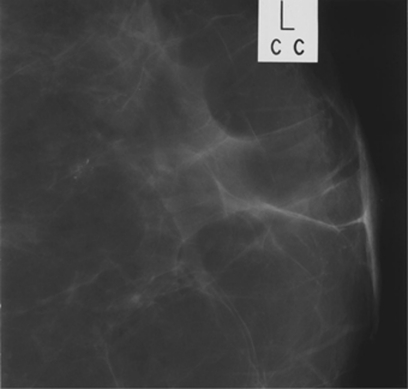
Indeterminate (category 3) microcalcifications. A small cluster of slightly pleomorphic microcalcifications.

Grade 4 calcifications exhibit significant pleomorphism but do not have clear-cut malignant features. Grade 3 calcifications do not exhibit classical benign features and are mildly pleomorphic. Figures 1,2,3 illustrate typical cases of grade 3–5 microcalcifications.

Statistical analyses were performed using SAS version 9.2 software (SAS Institute Inc., Cary, NC, USA). Risk factors satisfying a *P*-value criterion of *P*<0.20 in univariate log binomial regression models were included into a multivariate log binomial regression model. A *P*-value <0.05 in the multivariate model was considered statistically significant.

### Consent

This study was conducted with institutional review board approval; informed consent was waived.

## Results

### Demographic data

Between January 1992 and December 2007, 2545 cases of screen-detected microcalcifications of at least grade 3 were assessed by biopsy (mean 153.4 lesions per year). The mean age of the patients was 57.7 years (s.d. 8.4 years, range 40–86 years). In 187 women (7.3%), there was a very strong family history of breast cancer, defined in our programme historically as having either a first-degree relative with breast cancer before the age of 50 years, or with bilateral breast cancers at any age, or two first-degree relatives with breast cancer at any age. The microcalcifications were in the left breast in 1118 (53.0%) and in the right side in 990 cases (47.0%). Though no imaging features of invasion were identified, a mass was palpable in the region of the mammographic abnormality in 137 cases (5.4%). The calcifications were detected in the first episode of screening (round 1) in 894 lesions (35.1%) and during the subsequent 2–15 rounds in the remaining 64.9%.

### Imaging features

The lesions were classified as grade 3 in 1215 lesions (47.7%), grade 4 in 719 lesions (28.3%) and grade 5 in 611 lesions (24.0%). The distribution of the calcifications is specified in 2086 cases. The microcalcifications formed a single cluster in 1357 cases (65.1%), multiple clusters in 224 cases (10.7%) and were scattered in 505 cases (24.2%). The mammographic extent of microcalcifications was specified in 2088 cases. This ranged from 2 to 430 mm with a mean value of 21.8 mm (s.d. 23.8 mm). In total, 1229 (58.9%) microcalcifications were ⩽15 mm and 859 (41.1%) were >15 mm in imaging extent.

### Final outcome

After a diagnostic workup, 1220 lesions (47.9%) were found to be malignant and 1325 (52.1%) non-malignant. This group includes 122 (4.8%) premalignant lesions, comprising lobular carcinoma *in situ*, atypical lobular hyperplasia and atypical ductal hyperplasia (ADH). The malignant lesions consisted of 809 cases of DCIS (31.8%) and 411 invasive cancers (16.1%), almost all associated with a component of DCIS.

The histological grade of the DCIS is available in 677 of the 809 cases. The DCIS was classified as high grade in 396 cases (58.5%), intermediate grade in 64 cases (9.5%), low grade in 209 cases (30.9%) and as other, special subtypes (e.g., papillary, spindle cell) in 8 cases (1.2%).

Among malignant lesion, high-grade DCIS was observed in 88.5% of radiological grade 3 lesions, 90.8% of grade 4 lesions and 87.2% of grade 5 lesions. These differences were not significant (*P*=0.23).

The histological extent of the DCIS was specified in 620 cases. The DCIS measured 1–15 mm in 236 cases (38.1%), 16–40 mm in 263 cases (42.4%) and larger than 40 mm in 121 cases (19.5%).

Of the invasive cancers, 87 (21.2%) were grade 1, 180 (43.8%) were grade 2, 58 (14.1%) were grade 3, whereas in 77 (18.7%) histological grade could not be assessed formally mostly due to small size. Information regarding grade was not provided in nine cases (2.2%). Among the 359 invasive cancers with available data, 69 (19.2%) were 1–2 mm in diameter on histology, 76 (21.2%) were 3–5 mm, 101 (28.1%) were 6–10 mm, 59 (16.4%) were 11–15 mm, 28 (7.8%) were 16–20 mm, 24 (6.7%) were 21–50 mm (T2) and 2 cancers (0.6%) were >50 mm (T3).

Information regarding axillary staging is available in 352 of 411 invasive cancers. Nodal metastases were detected in 55 cases (15.6%). Of these cases, 25 had only one positive node, 22 had two to four positive nodes and 8 cases had more than four positive nodes.

### Cancers diagnosed subsequently

Eleven other women have developed breast malignancies, 2 DCIS, 9 invasive cancers, after non-malignant biopsies. Upon review, 7 of the 11 cases had benign assessment results for the microcalcifications, but later developed true interval cancers, affecting the ipsialteral breast in 4 cases and the contralateral breast in the other 3 cases.

The details of the remaining four cases are as follows: in two women, core biopsies of the microcalcifications led to a diagnosis of ADH. These women were subsequently diagnosed with DCIS. One was recalled early and the diagnosis was made at the subsequent screen 1 year later. The second woman declined further invitations for screening. Her DCIS was diagnosed 7.6 years later. Current assessment protocols would have recommended immediate diagnostic open biopsy in both these women.

The third woman had grade 3 microcalcifications, assessed at the time by FNA biopsy and interpreted as showing benign changes. She was later diagnosed with invasive cancer. On review, this is classified as a missed interval cancer. Under current protocols she would be assessed by vacuum-assisted core biopsy rather than by FNA.

The fourth woman had two clusters of microcalcifications at screening. After a non-diagnostic cytology result, she proceeded to open biopsy. This was directed at one cluster of microcalcifications and the histology of that lesion was benign. The other cluster was not excised. It was later found to include an invasive cancer. Current protocols would require vacuum-assisted core biopsy of this case.

### Predictors of malignancy – univariate analysis

Table 1 shows the proportion of malignancy for the variables of interest, and Table 2 outlines the univariate analysis of these findings. Women with a strong family history of breast cancer had a malignant lesion in 101 of 187 cases (54.0%), compared with 47.5% (1119 of 2358) of women without such a history. The relative risk (RR) was 1.1, not reaching statistical significance (0.068).

The proportion of lesions found to be malignant varied with age, being 41.7% in 40–49 year olds, 48.1% for women in their 50's, 48.3% for those in their 60's and 56.5% for those ⩾70 years. The RRs between the different age groups varied between 1 and 1.36, the differences being significant, as shown in Table 2. The biggest difference was a RR of 1.36 between women ⩾70 years and women younger than 50. The differences between women in their 50's *vs* women in their 60's, the target age group for our programme, were not significant (RR=1.0).

Among lesions detected in the first episode of screening 40.6% (363 of 894) proved to be malignant, whereas 51.9% (857 of 1651) of microcalcifications assessed in subsequent screening rounds were malignant. Compared with lesions detected at the first screen, the RR for detection at screening rounds 2–5 was elevated significantly at 1.29, and was 1.25 for lesions detected in rounds 6 or later. The rates of malignancy were, however, not significantly different for lesions assessed in rounds 2–5 *vs* later rounds.

Among the 137 women with a significant palpable mass corresponding to the mammographic lesion, 120 (87.6%) proved to have a malignancy compared with 45.7% of other women. Lesions associated with a palpable mass had a significantly higher RR for malignancy at 1.9 (*P*<0.0001). The majority of the lesions with a palpable mass (70.8%) had highly suspicious imaging features (grade 5). Lesions with grade 5 imaging features and a palpable mass proved to be malignant in 99.0% of cases.

The likelihood of malignancy varied significantly with the radiological grade of the calcifications. Among grade 3 lesions, 289 of 1215 (23.8%) were malignant, the corresponding figures were 373 of 719 (51.9%) for grade 4 lesions and 558 of 611 (91.3%) for grade 5 lesions. Compared with grade 3 lesions, the RRs for malignancy were 2.18 for grade 4 lesions and 3.84 for grade 5 lesions. These differences were significant, as was the difference between grade 4 and grade 5 lesions with the RR=1.78 (*P*<0.0001).

The rate of malignancy was 40.0% (543 of 1357) for cases with a single cluster of microcalcifications, 50% (112 of 224) for those with multiple clusters and 60.0% (303 of 505) for those with dispersed microcalcifications. Compared with women who had only one cluster of microcalcifications, the RR of malignancy for women with multiple clusters was significantly raised at 1.25. The RR was 1.5 for women with dispersed microcalcifications. The RR of malignancy was significantly higher at 1.20 for women with dispersed calcifications compared with those with multiple clusters of microcalcifications.

**Figure 4:**
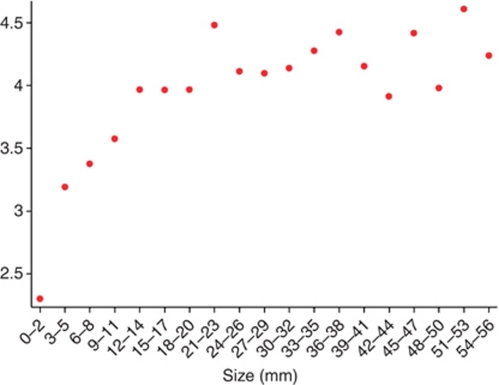
Scatter plot of log probability of malignancy (*y* axis) *vs* mammographic extent of microcalcifications. Because the relationship between size and log of the probability of malignancy is not linear, the appropriate statistical analysis was for size to be categorised as ⩽15 mm or >15 mm rather than as a continuous variable.

The likelihood of malignancy increased with the extent of the lesion on imaging. The rate of malignancy was 26.9% for lesions under 10 mm, 44.4% for those 10–19 mm, 59.2% for lesions 20–29 mm, 65.3% for lesions 30–39 mm, 68.3% for lesions 40–49 mm and 65.4% for lesions larger than 50 mm. An analysis of the log of probability of malignancy and mammographic extent of microcalcifications showed a step-wise increase after 15 mm and after 40 mm (Figure 4). Using these cut offs, the rate of malignancy was 424 of 1229 (34.5%) for lesions ⩽15 mm on imaging, 360 of 588 (61.2%) for lesions 16–40 mm and 181 of 271 (66.8%) for lesions >40 mm. Compared with microcalcifications ⩽15 mm in extent, the RR of malignancy was 1.8 for larger lesions. The differences were significant *P*<0.01.

### Multivariate analysis

The results of the multivariate analysis are shown in Table 3. The following factors were retained as independent predictors of malignancy: radiological grade, episode of screening, mammographic extent of microcalcifications and association with a palpable mass. Strong family history for breast cancer, age group and the distribution of calcifications lost significance in the multivariate analysis.

In the multivariate model, radiological grade had the largest effect. Compared with grade 3 lesions, grade 4 microcalcifications had a 2.2-fold higher RR, whereas the RR for grade 5 lesions was 3.3. The RR of grade 5 *vs* grade 4 microcalcifications was 1.5, also significant.

Compared with women whose microcalcifications were detected at the first screening episode, those detected in the subsequent 2–5 rounds had a higher likelihood of malignancy with a RR of 1.4. The RR was slightly higher at 1.5 for microcalcifications detected in rounds 6 and later but this difference did not reach statistical significance.

The extent of the microcalcifications was another independent predictor of malignancy. Compared with lesions ⩽15 mm, those >15 mm were 1.3 times more likely to be malignant (*P*<0.0001).

The presence of a palpable mass associated with the mammographic abnormality, although found in only 5.3% of patients, increased the RR to 1.1, which was significant (*P*=0.01).

## Discussion

In this large series of 2545 microcalcifications biopsied in the setting of an accredited population-based breast cancer-screening programme, 47.9% of the lesions biopsied were malignant, of which one-third were invasive cancers. This series has focused on cases in which the only mammographic abnormality was microcalcifications, without associated masses. Radiological grade was the most important independent predictor of malignancy, followed by detection after the first episode of screening, mammographic extent of microcalcifications and association with a clinically palpable mass.

### The strengths and limitations of radiological grading for microcalcifications

This analysis establishes the discriminative value of the radiological grading scheme adopted by the RANZCR in stratifying microcalcifications into risk groups with significantly different likelihoods of malignancy. This grading system has been in use at our centre for the last 23 years and is used throughout Australia, New Zealand and part of Europe. The positive predictive value (PPV) was 23.8% for grade 3 lesions, 54.5% for grade 4 lesions and 92.1% of grade 5 lesions (*P*=0.0001) with corresponding RRs of 2.2 for grade 4 lesions and 3.3 for grade 5 lesions when grade 3 lesions are the frame of reference.

The risk stratification by radiological grade is clinically helpful in counselling women. In particular, the high PPV of category 5 radiology, signifies a greater than 90% chance of malignancy, mandating careful exclusion of malignancy. However, even among grade 5 lesions, almost 10% of cases were ultimately shown to be non-malignant histologically. This observation reiterates the necessity to evaluate all suspicious microcalcifications by tissue biopsy before definitive treatment, as imaging alone is not a sufficiently robust determinant of malignancy.

The PPVs of the different lesion grades in this system are different and generally higher than the corresponding figures reported using the BIRAD system, underscoring the lack of equivalence of the two systems. Recent studies indicate a broad range of experiences in the PPV for the BIRAD 4 and 5 categories, reporting a range of 17–53% for BIRAD category 4 and 44–91% for category 5 lesions (Gulsun *et al*, 2003; Lazarus *et al*, 2006; Bent *et al*, 2010). The higher end of this range is similar to the 92.1% PPV of a grade 5 classification in our system.

Although the radiological grade successfully stratified microcalcifications into groups with different risks of malignancy, the radiological grade had no bearing on the histological grade of the underlying DCIS. Thus, regardless of the radiological grade, between 87 and 91% of DCIS found as a result of assessing screen-detected microcalcifications were found to show comedo necrosis. It is the necrotic contents of the involved ducts that undergo dystrophic calcification, and the presence of extensive comedo necrosis excludes a diagnosis of low-grade DCIS (Silverstein and Buchanan, 2003), explaining the over-representation of higher grades of DCIS in this setting. Our observations are consistent with other reports confirming that radiological grade does not predict DCIS grade reliably (Hermann *et al*, 1999; Dinkel *et al*, 2000; Slanetz *et al*, 2001; Stomper *et al*, 2003).

### The tradeoff between high cancer detection and the morbidity of a high recall rate

Although microcalcifications represent an early sign of an underlying breast malignancy, in the setting of population screening, the aim of cancer detection has to be balanced against the morbidity associated with investigation of non-malignant processes. As microcalcifications are a common finding on screening mammograms, stringent selection criteria and reasonable thresholds for recall are required to limit unnecessary investigations of large numbers of women.

Nationally organised population-based breast cancer-screening programmes, such as those implemented in Europe and Australia, have set accreditation standards to mitigate the unintended harms associated with screening of asymptomatic women and to reduce the rate of invasive investigations and anxiety for the participants. In our programme, the proportion of women recalled for assessment after their first round of screening should be <10% and <5% for those returning in subsequent rounds (BreastScreenAustralia, 2005). This audit of over 2500 microcalcifications assessed at our centre demonstrates that 47.9% of the biopsy cases proved to be malignant, whereas premalignant lesions were found in a further 4.8% of the women. This is a higher rate of malignancy than reported by some investigators, where the rate of malignancy for microcalcifications has been in the region of 25–30% (Kuzmiak *et al*, 2006; Lazarus *et al*, 2006; Burnside *et al*, 2007; Bent *et al*, 2010). Our findings are similar to the experience of the Nottingham group, who used a similar design and radiological grading scheme and found a 45% rate of malignancy (Burrell *et al*, 1996). In addressing the variation in the reported rates of malignancy, we note that the present series has focused on microcalcifications that were biopsied. Microcalcifications recalled, but cleared after further imaging workup, are not included. Their inclusion would have reduced the rate of malignancy significantly. A wider audit to include all cases is of interest and is planned for the future, but is outside the scope of the present series.

### Importance of microcalcifications in the diagnosis of early-stage breast cancer

The detection of small cancers, defined as those ⩽15 mm in diameter on histology, is an important indicator of screening quality. Our national accreditation standards mandate that at least 25 screen-detected cancers per 100 000 women screened should be ⩽15 mm (BreastScreenAustralia, 2005). Our data demonstrate that in addition to the strong association with high grade DCIS, the assessment of screen-detected microcalcifications is an important method of detecting invasive cancers, including small invasive cancers. In this series, 32.6% (235 of 721) of malignant cases, representing 16.1% of the entire cohort of lesions biopsied, constituted invasive cancers. This is despite the fact that our inclusion criteria limited the study to cases where the radiological abnormality was classified as only microcalcifications, without other associated imaging abnormalities. As detailed earlier, 85% (181 of 213) of the invasive cancers were under 16 mm in diameter. Despite their small size, only 21.2% of these cancers were grade 1, and 15.6% of them had metastasised to lymph nodes. The significant association of grade 3 cancers with DCIS was documented by Chagpar *et al* (2009) and evidence has been presented that when invasive malignancies present as calcifications, the invasive cancers are often HER2 positive, a feature that has been associated with poorer prognosis in prior studies (Gajdos *et al*, 2002; Seo *et al*, 2006).

Molecular studies of precursor lesions of breast cancer show a dichotomous pattern, grouping low-grade DCIS with low-grade invasive cancers, separate from the pairing of higher grade DCIS with grade 3 invasive cancers along a different pathway (Simpson *et al*, 2005). It appears that the assessment of screen-detected microcalcifications permits preferential diagnosis of higher grades of DCIS and of biologically more aggressive subsets of invasive cancer with which they are associated. In this context, the assessment of mammographic microcalcifications represents a valuable opportunity for altering the natural history of these cancers and reducing mortality from breast cancer.

### Concerns of over diagnosis

One of the criticisms of breast cancer-screening programmes is that a proportion of cancers detected through screening mammography may have been indolent and may never have presented clinically during the woman's lifetime, had they not been detected by screening. To the extent that these women face the morbidity and anxiety associated with the diagnosis and treatment of breast cancer, the diagnosis of these cancers is regarded as one of the negative consequences of screening. As there are presently no reliable means of predicting at the individual level which cancers are destined to progress and which will remain indolent, the estimates of the magnitude of such over-diagnosis are based on statistical inferences, with a rather wide range of estimates spanning zero to over 30% of cancers (National Breast Ovarian Cancer Centre, 2010). Nevertheless, the diagnosis of DCIS is included in these calculations and accounts for much of the allegedly over-diagnosed malignancies. We note that it has been established that DCIS shares the same predisposing factors as invasive breast cancer (Longnecker *et al*, 1996; Kerlikowske *et al*, 1997, 2003; Trentham-Dietz *et al*, 2000; Wohlfahrt *et al*, 2004) and that women with a diagnosis of DCIS are at 10 times greater risk for the future development of invasive breast cancer (Betsill *et al*, 1978; Page *et al*, 1995; Ernster *et al*, 2002). Recent trials, conducted in the era of screen-detected DCIS, have demonstrated that within 4–5 years after treatment of DCIS by local excision alone, women had an 8% rate of developing invasive breast cancer in the same region of the breast. These invasive cancers were more likely to be high grade than low grade (Kerlikowske *et al*, 2003). Treatment of the DCIS by the addition of radiation therapy or Tamoxifen halved the risk of development of invasive cancer (Fisher *et al*, 1999; Houghton *et al*, 2003). The relatively rapid time frame for the development of invasive cancer suggests that screen-detected DCIS is not a trivial process, but a non-obligate precursor of invasive breast cancer, and as such represents an important opportunity for medical intervention. Indirect support for this view has emerged from recent biological research, elucidating common defects in the molecular genetics of *in situ* and invasive breast cancers (Stratton *et al*, 1995; Castro *et al*, 2008; Wiechmann and Kuerer, 2008).

Rather than upholding the validity of concerns for over-diagnosis of an indolent process, our findings support the view that the assessment of microcalcifications leads to the detection of a significant number of biologically significant invasive cancers and preferentially detects higher grades of DCIS (Franceschi *et al*, 1990; Evans and Blanks, 2002; Stomper *et al*, 2003; Kuzmiak *et al*, 2006).

We found mammographic extent of microcalcifications to be an independent predictor of malignancy. The analysis of the relationship between the mammographic extent of microcalcifications and malignancy revealed an apparent stepwise increase in the likelihood of malignancy, with the increases occurring in the regions of 15 and 40 mm. We note that these are the cut offs used in the Van Nuys Prognostic index, but in that setting applied to the histological as opposed to the imaging extent of the lesion (Silverstein and Lagios, 2010). Even among lesions <10 mm in extent 16.9% proved to be malignant, emphasising the need for vigilance.

Our results highlight the value of physical examination. Although a palpable mass was associated with the imaging abnormality in only a minority of the women, this finding was highly predictive of a malignant diagnosis. This association has implications for the design of screening programmes, as inclusion of physical examination in the assessment process provides an additional safety net in avoiding false negative assessment results.

The observed significant difference in the rate of malignancy when the microcalcifications were detected after the first episode of screening is likely due to the fact that recall in subsequent rounds signifies interval change, either through the new development of microcalcifications or significant interval progression between screening mammograms. This observation is accounted for by the fact that the women are older in subsequent rounds, and age is a risk factor for the development of malignancy. But also, since in subsequent rounds the radiologists have access to the prior images taken at preceding rounds, they are in a better position to detect true interval changes. Consequently stable microcalcifications, likely to be benign, are apt not to be recalled for assessment.

Among the factors that were not retained in the final model, the findings in relation to age were of interest. The rate of malignancy among the women younger than the target age group of 50–69 years was 41.7%. This was a significantly lower rate than the 48.05% within the target age group. Women older than 70 years had the highest rate of malignant diagnoses at 56.5%. Although age was not an independent predictor of malignancy when other variables were included, these observations may be of interest in the ongoing debate regarding the optimal age limits for screening.

Family history was reported in relatively few women, and while women with a strong family history had a RR of 1.14, this effect was not significant in the univariate or indeed the final multivariate model. These findings may contribute to the discussions regarding the frequency of screening, particularly for women with a family history of breast cancer.

## Conclusions

The assessment of screen-detected microcalcifications represents an important opportunity to diagnose DCIS and biologically significant invasive breast cancer at an early stage. Although the thresholds for recall and biopsy of microcalcifications may vary between centres, at our institution almost half of lesions biopsied proved to be malignant. This audit demonstrates a relatively high specificity of biopsy assessment, ensuring that the morbidity associated with unnecessary biopsy is reduced, while achieving high cancer detection rates.

The demonstration of the stepwise increase in the likelihood of malignancy between lesions of different imaging grade and the significant differences between these grades validates the grading scheme adopted by the Royal Australian and New Zealand College of Radiologists.

## Figures and Tables

**Table 1:** Descriptive statistics for 2545 screen-detected biopsied microcalcifications

**Variable**	**Level**	**Total number**	**No. of malignant cases**	**% Malignant cases**
Strong family history of breast cancer	No	2358	1119	47.5
	Yes	187	101	54.0
				
Age group (years)	<50	360	150	41.7
	50–59	1180	567	48.1
	60–69	789	381	48.3
	70+	216	122	56.5
				
Screening round	1	894	363	40.6
	2–5	1290	674	52.3
	6+	361	183	50.7
				
Palpable mass	No	2408	1100	45.7
	Yes	137	120	87.6
				
Radiological grade	3	1215	289	23.8
	4	719	373	51.9
	5	611	558	91.3
				
Distribution of calcifications	Missing	459	262	57.1
	Single cluster	1357	543	40.0
	Multiple clusters	224	112	50.0
	Dispersed	505	303	60.0
				
Imaging extent	Missing	457	255	55.8
	15 or less	1229	424	34.5
	>15	859	541	63.0
				

**Table 2:** Risk factors for malignancy – univariate analysis

**Variable**	**Relative risk**	**Lower 95% CI**	**Upper 95% CI**	***P*-value**
Strong family history: yes *vs* no	1.14	0.99	1.31	0.07
				
*Age group (years)*				0.01
50–59 *vs* <50	1.15	1.01	1.32	0.04
60–69 *vs* <50	1.16	1.01	1.34	0.04
70+ *vs* <50	1.36	1.14	1.61	<0.01
60–69 *vs* 50–59	1.01	0.92	1.10	0.92
70+ *vs* 50–59	1.18	1.03	1.34	0.02
70+ *vs* 60–69	1.17	1.02	1.34	0.03
				
*Screening round*				<0.01
2–5 *vs* 1	1.29	1.17	1.41	<0.01
6+ *vs* 1	1.25	1.10	1.42	0.00
6+ *vs* 2–5	0.97	0.87	1.09	0.60
				
Palpable mass: yes *vs* no	1.92	1.78	2.07	<0.01
				
*Radiological grade*				<0.01
4 *vs* 3	2.18	1.93	2.47	<0.01
5 *vs* 3	3.84	3.46	4.26	<0.01
5 *vs* 4	1.76	1.63	1.90	<0.01
				
*Distribution*				<0.01
Multiple *vs* single	1.25	1.08	1.45	<0.01
Dispersed *vs* single	1.50	1.36	1.65	<0.01
Dispersed *vs* multiple	1.20	1.03	1.39	0.02
				
Size >15 *vs* 15 or less	1.83	1.66	2.00	<0.01

Abbreviations: CI=confidence interval; DCIS=ductal carcinoma *in situ.*

In order to identify risk factors for malignancy (DCIS or invasive), univariate log binomial regression models were fitted to the data. Relative risks and 95% CIs for each of the risk factors are shown in the table below.

**Table 3:** Multivariate model for malignancy. A multivariate log binomial regression model including all predictors was fitted to the data

**Variable**	**Relative risk**	**Lower 95% CI**	**Upper 95% CI**	***P*-value**
Strong family history: yes *vs* no	1.12	0.99	1.28	0.08
				
*Age group (years)*				0.14
50–59 *vs* <50	1.03	0.89	1.18	0.72
60–69 *vs* <50	0.96	0.82	1.11	0.58
70+ *vs* <50	1.11	0.93	1.32	0.24
60–69 *vs* 50–59	0.93	0.85	1.02	0.14
70+ *vs* 50–59	1.08	0.95	1.22	0.23
70+ *vs* 60–69	1.16	1.02	1.32	0.03
				
*Screening round*				<0.01
2–5 *vs* 1	1.42	1.27	1.58	<0.01
6+ *vs* 1	1.54	1.35	1.77	<0.01
6+ *vs* 2–5	1.09	0.98	1.21	0.11
				
Palpable mass: yes *vs* no	1.13	1.03	1.24	0.01
				
*Radiological grade*				<0.01
4 *vs* 3	2.20	1.94	2.51	<0.01
5 *vs* 3	3.26	2.90	3.65	<0.01
5 *vs* 4	1.48	1.36	1.61	<0.01
				
*Distribution*				0.52
Multiple *vs* single	0.96	0.84	1.10	0.57
Dispersed *vs* single	1.03	0.94	1.13	0.51
Dispersed *vs* multiple	1.07	0.95	1.22	0.28
				
Size >15 *vs* 15 or less	1.29	1.17	1.44	<0.01
